# Hereditary Spherocytosis: Unravelling the Diagnostic Challenges

**DOI:** 10.7759/cureus.70308

**Published:** 2024-09-27

**Authors:** Kishor M Khillare, Bhumika Vaishnav, Nikhil I Doshi, Ruchitha Pailla, Aniruddh Wadivkar

**Affiliations:** 1 General Medicine, Dr. D. Y. Patil Medical College, Hospital and Research Centre, Dr. D. Y. Patil Vidyapeeth (Deemed to be University), Pune, IND

**Keywords:** eosin-5'-maleimide, hereditary spherocytosis, osmotic fragility test, sickling test, spleenomegaly

## Abstract

Hereditary spherocytosis (HS) is a genetic disorder characterized by the presence of spherocytes, which are abnormally shaped red blood cells, leading to hemolytic anemia. While HS is not uncommon in hematology, it can present significant diagnostic and therapeutic challenges in its late stages, particularly when complicated by severe cholestasis. We report a case of a 48-year-old male presenting with jaundice and abdominal pain, initially diagnosed with cholecystolithiasis and moderate splenomegaly. Subsequent investigations confirmed HS through clinical observation and eosin-5'-maleimide (EMA) binding by flow cytometry. The patient exhibited severe jaundice (total bilirubin: 19.58 mg/dL) but no anemia. The complexity of his condition necessitated a multidisciplinary approach involving hematologists and gastroenterologists along with general medicine. This case underscores the importance of considering HS in the differential diagnosis of cholestasis and highlights the need for comprehensive diagnostic strategies to manage such complications effectively.

## Introduction

Hereditary spherocytosis (HS) is an inherited form of hemolytic anemia caused by defects in the proteins of the red blood cell (RBC) membrane, resulting in the production of spherocytes [1]. These spherocytes exhibit reduced flexibility and are more susceptible to hemolysis as they traverse the spleen. Hereditary spherocytosis is primarily transmitted through an autosomal dominant inheritance pattern, though autosomal recessive forms are also observed [2]. It predominantly affects infants and children, with clinical manifestations ranging from mild to severe anemia, jaundice, and splenomegaly.

The pathophysiology of HS involves mutations in genes encoding RBC membrane proteins such as ankyrin 1, band 3, α-spectrin, β-spectrin, and protein 4.2 [3]. These mutations compromise the structural integrity of the RBC membrane, causing the cells to adopt a spherical shape. The spleen, which filters out abnormal cells, subsequently destroys these spherocytes, leading to hemolytic anemia.

HS patients often present with jaundice, pallor, fatigue, and splenomegaly. The diagnosis is typically made based on clinical findings, family history, and laboratory tests including a complete blood count (CBC), reticulocyte count, peripheral blood smear, and an osmotic fragility test (OFT). Flow cytometry using eosin-5'-maleimide (EMA) binding is a sensitive diagnostic test for HS, detecting abnormalities in RBC membrane proteins [4].

Complications of HS include aplastic crises, typically triggered by parvovirus B19 infection, and hemolytic crises due to increased hemolysis. Cholelithiasis is another common complication due to chronic hemolysis leading to hyperbilirubinemia. Cholecystitis, an inflammation of the gallbladder, can occur if gallstones obstruct the cystic duct, causing significant clinical challenges. Management of HS focuses on relieving symptoms and preventing complications. This includes folic acid supplementation, blood transfusions in severe cases, and splenectomy in patients with severe or refractory HS including recurrent hemolytic crisis or severe aplastic crisis [4].

This case report includes the presentation, diagnosis, and management of a 48-year-old male with HS complicated by cholecystitis, highlighting the diagnostic challenges and treatment strategies employed.

## Case presentation

A 48-year-old male presented to a tertiary care center with complaints of jaundice and abdominal pain of one-year duration. The patient had been experiencing dull, non-radiating pain in both the right and left hypochondriac region, accompanied by vomiting and loose stools for the past year. Initial investigations at a local hospital revealed elevated bilirubin levels. An abdominal ultrasound showed cholecystolithiasis, gallbladder sludge, and moderate splenomegaly with normal liver size, shape, echo pattern, and normal portal vein, prompting his referral to our facility.

The patient had a medical history of recurrent yellowish discoloration of eyes and nails; he also had on-and-off pain in the abdomen in the right hypochondriac region that had been non-radiating for a year. To address these complaints, the patient was hospitalized many times, and recently he was advised for cholecystectomy but remained undiagnosed for persistent jaundice and hypersplenism for such a long time. He had no family history of genetic disorders. He was a chronic alcoholic but a non-smoker. On admission, a physical examination revealed yellowish discoloration of the sclera (icterus) and skin, with no pallor, cyanosis, clubbing, or lymphadenopathy. The abdominal examination revealed mild tenderness in the left hypochondrial region and an enlarged spleen (splenomegaly). Routine biochemical investigations were within limits including blood coagulation (international normalized ratio, or INR, was 0.90 and prothrombin time was 12.24 seconds) and serum albumin at 4.4g/dL. The liver function test (LFT) was deranged (Table 1).

**Table 1:** Blood investigations on admission and during discharge HB: hemoglobin, TLC: total leukocyte count, RBC: red blood cell, RDW: red cell distribution width, AST: aspartate aminotransferase, ALT: alanine aminotransferase, ALP: alkaline phosphatase, LDH: lactate dehydrogenase, TIBC: total iron binding capacity, ANA-IF: antinuclear antibody by immunofluorescence

Investigations	Admission Result	Discharge Result
HB	13.1g/dL	13.2 g/dL
TLC	10,300/µL	8600/µL
Platelets	2,22,000/µL	2,30,000/µL
RBC count	4.38 x 10^6^/µL	4.26 x 10^6^/µL
Hematocrit	37.2%	30.8%
RDW	18.7%	20.1%
Sodium	138 mmol/L	139 mmol/L
Potassium	3.68 mmol/L	4.1 mmol/L
Urea	18 mg/dL	19 mg/dL
Creatinine	0.58 mg/dL	0.60 mg/dL
Bilirubin total	19.58 mg/dL	1.50 mg/dL
Bilirubin conjugated	6.8 mg/dL	0.80 mg/dL
Bilirubin unconjugated	12.78 mg/dL	0.60 mg/dL
AST	51 u/L	47 u/L
ALT	132 u/L	67 u/L
ALP	282 u/L	100 u/L
Reticulocyte count	3.6%	Not repeated
LDH	264 u/L	Not repeated
Indirect Coombs test	Negative	Not repeated
Direct Coombs test	Negative	Not repeated
Iron	91 mcg/dL	Not repeated
TIBC	240 mcg/dL	Not repeated
Transferrin saturation	37.9%	Not repeated
Ferritin	1152.8 ng/mL	Not repeated
Osmotic fragility test	Mild increase	Not repeated
Hb electrophoresis	Normal	Not repeated
Sickling test	Negative	Not repeated
ANA-IF	Negative	Not repeated

To confirm the OFT report, EMA binding by flow cytometry was done, which showed a 19.5% reduction in the mean fluorescence intensity (MFI), suggestive of HS.

The patient was given analgesics to manage abdominal pain and antiemetics for vomiting. Intravenous (IV) fluids were administered to maintain hydration. The patient was started on 5 mg of folic acid daily to support red blood cell production. Regular monitoring of hemoglobin and bilirubin levels was done to assess the severity of hemolysis and effectiveness of treatment. Broad-spectrum antibiotics were administered to prevent infection associated with cholecystitis. Furthermore, an elective laparoscopic cholecystectomy was performed by a gastrointestinal surgeon after stabilizing the patient to prevent recurrent cholecystitis and gallstone formation. Postoperative magnetic resonance cholangiopancreatography (MRCP) revealed no obstructive biliary dilatation, intraluminal filling defects, or pancreatitis, but showed localized fluid collection in the gallbladder fossa and a mild irregularity in the right lateral wall of the common hepatic duct. The patient was discharged from the hospital after showing significant improvement in clinical symptoms and biochemical parameters. The treatment effectively stabilized the patient’s condition, as evidenced by near normalized LFTs and improved hematological indices.

The patient was advised to receive immunizations against encapsulated organisms (*Haemophilus influenzae*, *Streptococcus pneumoniae*, and *Neisseria meningitidis*) in anticipation of a possible splenectomy. At the time of discharge, the patient received counselling about the chronic nature of HS, potential complications, and the importance of adherence to follow-up and treatment plans. Also, the patient and his family were advised to undergo genetic counselling to assess the risk of HS in family members and discuss reproductive options.

## Discussion

Based on symptoms, there are three types of hereditary spherocytosis: mild HS, moderate HS, and severe HS. HS can commonly present with anemia, generalized weakness, fatigue, jaundice, abdominal pain, gallstones, or splenomegaly. Mild HS is diagnosed in about 20%-30% of patients, where patients may not have anemia or may have only mild reticulocytosis, with the disorder remaining undetected until adulthood. Moderate disorder accounts for 65%-70% of cases, where patients have anemia, elevated reticulocyte counts, and high serum bilirubin levels, and may require occasional RBC transfusions. Severe disorder is seen in approximately 4%-6% of cases and is characterized by significant hemolysis, anemia, hyperbilirubinemia, and splenomegaly, requiring regular red blood cell transfusions [5,6].

This case highlights the challenges in diagnosing and managing HS, especially when complicated by a condition like cholecystitis. The patient's presentation, characterized by severe jaundice in the absence of anemia and the presence of gallstones, made the diagnosis particularly difficult until the patient reached our tertiary care center. The patient visited multiple clinics but remained undiagnosed for HS. HS was ultimately confirmed using EMA binding via flow cytometry, a diagnostic test for detecting defects in RBC membranes [7,8]. When the diagnosis of HS is uncertain, tests such as EMA binding or the osmotic fragility test are valuable. This flow cytometric test measures the fluorescent intensity of intact red cells labeled with EMA, which reacts covalently with Lys-430 on the first extracellular loop of the Band 3 protein. The N-terminal cytoplasmic domain of Band 3 interacts with ankyrin and protein 4.2, which crosslinks with the spectrin-based cytoskeleton and stabilizes the membrane lipid bilayer. The deficiency or abnormality of Band 3 may result in decreased fluorescence. This is seen in HS [9,10].

The treatment plan involved multiple strategies to address the patient’s symptoms and underlying conditions. Symptomatic management with analgesics and antiemetics, along with IV fluids improved the patient's immediate condition. Folic acid supplementation was given to support erythropoiesis.

In managing the hemolysis associated with HS, regular monitoring of hemoglobin, reticulocyte count, and bilirubin levels was essential to track the severity of hemolysis and the effectiveness of the treatment. Recent guidelines have updated the approach to managing HS, particularly regarding splenectomy in conjunction with cholecystectomy. It is now recognized that splenectomy carries long-term risks, and there is a greater emphasis on balancing these risks with the benefits of retaining some splenic function. Advances in diagnostic tools, such as the EMA binding test, have improved our ability to diagnose HS accurately, although these tests have their limitations. Practical case management has highlighted the complexity of HS when complicated by severe cholestasis. Effective treatment involves a multidisciplinary approach, including stabilization, cholecystectomy, and patient education on the chronic nature of HS and the need for follow-up. These developments underscore the importance of personalized care and careful consideration of the risks and benefits of surgical interventions in HS [5].

## Conclusions

This case highlights the complexity of managing hereditary spherocytosis, particularly when complicated by cholecystitis. Effective treatment required a multidisciplinary approach involving symptomatic management, infection control, and planning for elective cholecystectomy. Regular monitoring and patient education were crucial in addressing the chronic and variable presentation of HS. This case underscores the importance of considering HS in patients with unexplained jaundice and the critical role of comprehensive diagnostic and treatment strategies in managing such complex cases.
